# Evaluation of factors associated with medical male circumcision in South Africa: A case-control study

**DOI:** 10.4102/phcfm.v14i1.3500

**Published:** 2022-10-31

**Authors:** Sylvester O. Okhue, Robert J. Mash

**Affiliations:** 1 Department of Family Medicine and Primary Care, Faculty of Medicine and Health Sciences, Stellenbosch University, Cape Town, South Africa

**Keywords:** disease prevention, HIV, male medical circumcision, primary health care, primary care, health promotion

## Abstract

**Background:**

The World Health Organization recommends medical male circumcision (MMC) to prevent human immunodeficiency virus (HIV). More research is needed in South Africa on factors influencing the uptake of MMC.

**Aim:**

To evaluate factors associated with uptake of MMC.

**Setting:**

Diepsloot, Johannesburg, South Africa.

**Methods:**

An observational case-control study. Cases (men attending a private general practice (GP) offering free MMC) were compared to controls (uncircumcised men attending a local shopping mall) for a variety of demographic, sociocultural and financial factors. Factors were analysed using bivariate and multiple-variable binary forward logistic regression with the Statistical Package for Social Sciences.

**Results:**

There were 350 cases and 350 controls. Four factors were associated with the uptake of MMC: being a student (adjusted odds ratio [AOR]: 6.29, 95% confidence interval [CI]: 2.29–17.26), attending a mainline Christian denomination (AOR 2.85, 95% CI: 1.39–5.78), speaking an African language other than Zulu (range of AORs: 2.5–6.8, *p* < 0.05) and being South African (AOR: 2.50, 95% CI: 1.58–3.96). MMC was associated with feeling susceptible to HIV, seeing it as a serious health problem and being encouraged by partners. Men who were sterilised, not sexually active and without symptoms of a sexually transmitted infection felt less susceptible. Other barriers included the pain of the procedure, indirect costs, anticipated impact on sexual activity, lack of information, cultural beliefs, embarrassment and access to health services.

**Conclusion:**

Disease prevention initiatives should take note of the factors associated with MMC in this community. Further qualitative studies should explore issues behind the factors identified and provide further insights.

**Contribution:**

This study helps to identify factors that health services should address when implementing medical male circumcision.

## Introduction

Human immunodeficiency virus (HIV) is one of the major health problems in low- and middle-income countries, with over 38 million people infected worldwide.^1^ It is estimated that 25 million people in sub-Saharan Africa are living with HIV, and 7.7 million are in South Africa.^2^ Combined, HIV and human papilloma virus (HPV) account for nearly 69% of all sexually transmitted diseases globally.^3^ Infection with HIV is associated with risk-taking behaviours, such as multiple sexual partners, unprotected sexual contact, injection drug use and anal intercourse.^3^

Studies conducted in the United States of America and Israel, where circumcision rates are high and HIV burden is relatively low, show an inverse association between circumcision and HIV acquisition.^4^ Medical male circumcision (MMC) reduces the female-to-male sexual transmission of HIV by 60%.^5^ It remains the only once-off intervention that can reduce the risk of HIV infection and be highly cost-effective.^5^ In experienced hands, this inexpensive procedure is very safe and can be performed at any age. The public health benefits include protection from urinary tract infections, HIV, HPV, syphilis, chancroid, penile and prostate cancer, phimosis, thrush and inflammatory dermatoses.^6^ Since 2007, the World Health Organization (WHO) and Joint United Nations Programme on HIV and AIDS (UNAIDS) have recommended voluntary MMC as a key component of HIV prevention in countries with a high prevalence and low levels of circumcision.^5^ As a result, 14 countries in East Africa and Southern Africa, including South Africa, were identified as priorities and programmes were initiated to expand the provision of MMC.^5^

The majority of HIV infections (at least 70%) in sub-Saharan Africa are thought to be a result of penetrative sexual intercourse.^7^ Physiologically, sexual contact (by its very nature) results in the penis or vagina becoming the main portals of entry. Guided by this realisation, the mainstays of HIV prevention strategies have been the ‘abstain, be faithful, use condoms’ (ABCs) approach, together with MMC.^7,8^

The introduction and expansion of highly active antiretroviral therapy (HAART) was associated with a sustained and profound population-level decrease in morbidity and mortality from HIV, as well as reduced transmission.^9^ The findings support the long-term effectiveness of HIV treatment both as a therapeutic and a preventive intervention. Evidence has shown that individuals on HAART with an undetectable viral load are very unlikely to transmit HIV to others.^9^ This, however, assumes that people living with HIV have access to HAART and are adherent to treatment.^9^ There is therefore still a place for MMC in the approach to prevention.

The United Nations report that 37% of men are circumcised globally.^10^ If 80% of South African men were circumcised by 2025, a total of 3.36 million HIV infections could be averted, which represents 22% of new HIV infections.^11^ So important is the potential preventive value of MMC that health services have actively promoted it.^12^ In South Africa, current rates of male circumcision are reported to vary from 35% to 43%, and traditional circumcision is performed as a rite of passage from boyhood to manhood in several ethnic groups, such as the AmaXhosa.^13^ South Africa represents a particularly interesting case study by virtue of its complex sociocultural context. It has multiple cultures, all with different health beliefs that may impact the uptake of circumcision.^13^ Male medical circumcision is also strongly associated with religious beliefs.^14^

To date, little research has been conducted to identify the demographic, sociocultural and financial factors that may contribute to an individual’s decision to have MMC.^13^ The key factors that are known to influence men’s decisions include a need to reduce the risk of HIV and sexually transmitted infections, the influence of sexual partners, desire for personal hygiene, a positive community perception of male circumcision and the person’s age.^15^ Specific costs related to accessing male circumcision services were reported as transport, materials for wound care and loss of days at work.^16^ Circumcision coverage in South Africa has been promoted using mass media, posters, billboards and automobiles with loudspeakers that drive through communities, especially when outreach services are planned. However, decisions are strongly influenced by sexual partners.^2,15^ The paucity of research, along with a need to develop in-depth understanding of circumcision practices in South Africa, provided the initial impetus for conducting a study that aimed to investigate what role demographic, sociocultural and financial factors have on the uptake of MMC in Diepsloot, Johannesburg, South Africa. The findings should help inform the design of public health and disease prevention initiatives that promote MMC.

## Methods

### Study design

This was an analytical observational case-control study that compared cases (men with MMC) and controls (men without MMC) and identified the factors associated with a decision to have MMC.

### Setting

This study was conducted in Diepsloot, a densely populated and impoverished township in the north of Johannesburg, South Africa. The population of the township was 138 329 as per 2011 census with the following population groups: black people (98%), mixed race people (0.2%), Asian people (0.1%), white people (0.2%) and other (1.5%).^17^ The township comprised a combination of fully subsidised housing, individually owned brick houses and shacks. There were nearly 4000 families living in backyard informal dwellings and an additional 6035 families living either in incomplete structures or temporary accommodation.^1^ Diepsloot has, as part of the Alexandra renewal project, accommodated a further 5000 families who now reside in the community. Diepsloot is severely economically disadvantaged and as a result exhibits many of the societal challenges that can be expected in high poverty areas.

The local clinics manage acute and chronic medical conditions. Emergency services are provided by the nearby public hospital. There is also provision for antenatal care and immunisation programmes. The private sector also manages acute and chronic cases, although chronic cases are less common as patients receive free services in the public sector. The private doctors refer cash patients to the nearby government hospitals and insured patients to the private hospitals.

Medical male circumcision is included in the service in both the public and private sectors. It is free in the government clinics, but it incurs a fee for service in the private sector. However, one private practice offered free MMC to the community as part of the government initiative.

### Study population

This study was conducted with men between the ages of 18 and 55 years living in Diepsloot, as this was the target group for MMC. Men who did not live in Diepsloot or had a traditional circumcision were excluded from the study.

### Sample size

Sample size for an unmatched case-control study was calculated using Epi Info. The following assumptions were made:

- 95% confidence intervals
- 80% power
- ratio of cases : controls 1:1
- expected odds ratio 1.7 with 31% of controls and 43% of cases exposed (based on those passing matriculation as a key educational factor^1^).

Using the Kelsey method, this gave a sample size of 243 in each group or 486 in total. It was assumed that five variables would be compared for their association with outcomes in the final multivariable analysis, and therefore an additional 10% was needed for each variable. This gave a final sample size of 364 cases and 364 controls, totalling 728 cases.^15^

### Sampling strategy

Cases were recruited from a local private practice, run by a local general practitioner, who offered free MMC to the community as part of the government initiative. This practice circumcised 50 people per day in the winter and 20 per day in the summer. People were recruited on a consecutive basis until the sample size was obtained, and only 11 men refused to consent.

Controls were recruited from the local shopping mall, which was used by all community members. Three research assistants were invited to help recruit controls, and the mall also provided a private more secluded space to obtain consent and complete the questionnaire. People meeting the inclusion and exclusion criteria were recruited on a random systematic basis as they entered the mall, whereby every third person was invited to participate until the sample size was obtained. All men who consented were then asked about their circumcision status, and those who were not circumcised were allocated to the controls. The exact ratio (i.e. every *n*th person) was determined by the time taken to consent and complete the questionnaire in privacy and the need to obtain the sample size. If someone refused to participate (23 people), then the next person was selected according to the systematic process. The reasons for refusal were not recorded.

### Data collection

The research focused on demographic, sociocultural and financial factors (Table 1). Demographic factors were defined as the characteristics of human populations, such as age and gender. Sociocultural factors were seen as the beliefs, customs and practices of community members that affected their behaviour. Financial factors were defined by employment, education and income.

**TABLE 1:** Factors measured in the study.

Demographic	Sociocultural	Financial
Age	Home language	Education
Gender	Religion	Employment
Marital status	Nationality	Income
Location or municipal ward	Beliefs and attitudes towards circumcision	-

A questionnaire was developed to measure these factors. Demographic and financial questions were derived from established questionnaires used in South Africa,^18^ and statements on beliefs and attitudes to MMC were derived from the literature.^19^ The draft questionnaire was validated for its content by a panel consisting of five people: two community leaders in Diepsloot, two local medical practitioners and the author’s supervisor. This combined expertise in both research design and the local community. The panel was asked to give feedback on the content of the questionnaire and whether all the relevant issues were included or any irrelevant issues needed to be excluded. They also gave feedback on whether the questions were clearly constructed and understandable in the local community and likely to give a valid answer. The questionnaire was modified based on feedback from the panel, and once finally approved, it was piloted. Piloting was conducted with typical respondents from the local community and intended to test the feasibility and acceptability of the questionnaire, such as how long it took, how it was interpreted and if any questions were problematic.

The questionnaire was translated by the Language Centre of Stellenbosch University and piloted with community members to ensure that it was easily understood in the local community. The languages were IsiZulu, IsiXhosa, Setswana and Tsonga.

Data were collected in 2021. The respondents completed the questionnaire in a private area of the general practice (GP). The research assistant took consent and was available to assist with the questionnaire if necessary. The questionnaire was completed by the respondents before having the circumcision, as the pain experienced after circumcision made it difficult to complete the questionnaire. In the mall, the research assistants took potential participants to a private and secluded area where they completed the questionnaire in a similar process. The researcher was assisted by three trained male research assistants. One assistant was a clinical associate from the researcher’s practice, and the other two assistants were contracted. They were all trained and held at least a basic degree and were proficient in the local languages.

### Data analysis

Data were captured in an Excel spreadsheet and checked for errors or omissions. The data were then analysed using Statistical Package for Social Sciences version 25. The supervisor, with support from the statistician at Stellenbosch University, assisted the researcher to perform the analysis.

Data were analysed by descriptive statistics with means and standard deviations (numerical data) or frequencies and percentages (categorical data). If not normally distributed, numerical data were described using medians and interquartile ranges (IQR).

Each independent variable was then tested for an association with cases or controls in a bivariate binary logistic regression. Associations with *p* < 0.2 were then included in a multivariable binary forward logistic regression. The results were presented as unadjusted and adjusted odds ratios with 95% confidence intervals.

### Ethical considerations

Ethical approval to conduct the study was obtained from the Health Research Ethics Committee of Stellenbosch University (ref. no. HREC2-2020-11877). Permission to conduct the research was obtained from the private GP and shopping mall (ref. no. S19/10/276).

## Results

**FIGURE 1:**
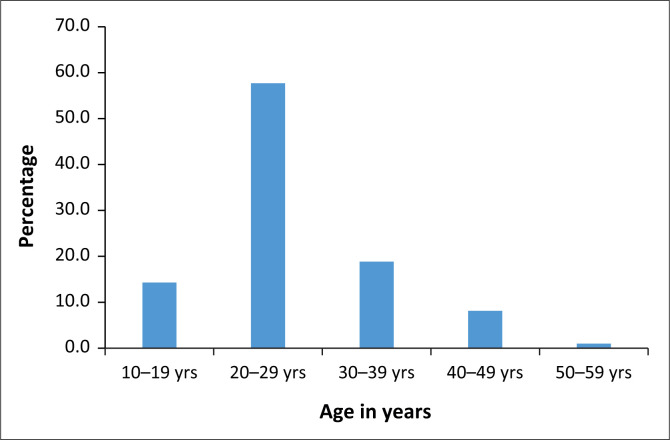
Age distribution of the study sample.

Table 2 describes key characteristics of the study sample as a whole. The 700 participants were mostly single, had education up to high school and were unemployed, of no particular religion and South African. There were a substantial number of immigrants as well, particularly from Zimbabwe. The predominant languages were Zulu and Ndebele. Very few people were married (5.9%), although 26% lived with a long-term partner. The median age was 25 years (IQR 25–27) for the cases and 26 years (IQR 26–27) for the controls. The age distribution is shown in Figure 1.

**TABLE 2:** Characteristics of the study sample (*N* = 700).

Variables	*n*	%
**Nationality**		
South Africa	411	58.7
Zimbabwe	126	18.0
Malawi	44	6.3
Lesotho	41	5.9
Eswatini	15	2.1
Mozambique	25	3.6
Botswana	22	3.1
Namibia	15	2.1
Unknown	1	0.0
**Marital status**		
Single	449	64.6
Partner	181	26.0
Married	41	5.9
Divorced	23	3.3
Unknown	6	0.2
**Highest qualification**		
None or some primary school	99	14.2
Completed primary school	153	21.9
Some high school	169	24.2
Completed high school	218	31.2
Certificate or diploma	51	7.3
Degree	9	1.3
Unknown	1	0.0
**Religion**		
None	574	83.9
Catholic	63	9.2
Protestant	2	0.3
Zionist	39	5.7
Muslim	5	0.7
Unknown	17	2.4
**Employment**		
Unemployed	290	41.8
Employed	224	32.3
Self-employed	73	10.4
Informal	47	6.7
Student	54	7.7
Other	5	0.7
Unknown	7	0.1
**Language**		
Zulu	153	21.9
Xhosa	76	10.9
Ndebele	143	20.5
Siswati	41	5.9
Sepedi	57	8.2
Setswana	40	5.7
Sotho	44	6.3
Tonga	37	5.3
Venda	23	3.3
Other	86	12.3
**Address**		
Extension 1	97	13.9
Extension 2	165	23.6
Extension 3	54	7.7
Extension 4	85	12.2
Extension 5	43	6.2
Extension 6	49	7.0
Extension 7	41	5.9
Extension 8	55	7.9
Extension 9	32	4.6
Extension 10	29	4.2
Extension 11	20	2.9
Extension 12	13	1.9
Extension 13	15	2.1

Table 3 presents the views of participants on various beliefs and attitudes related to MMC. The majority saw MMC as having health benefits, especially in terms of protecting them from HIV and HPV. The one worrying response was that the majority (70.2%) of respondents believed that MMC would still protect them from infection even when they did not use protection. The majority of respondents did not see MMC as against their culture and thought that their partner wanted them to have it. They did not anticipate any negative effects on sexual performance or sexual relationships. The majority of respondents had information about MMC and did not anticipate it being too expensive, painful or embarrassing. They were happy with the waiting times and opening hours of the clinic where they could get the MMC and saw it as a good investment of their time.

**TABLE 3:** The attitudes and beliefs of respondents (*N* = 700).

Statements	Strongly disagree		Disagree		Agree		Strongly agree		Unknown	
	*n*	%	*n*	%	*n*	%	*n*	%	*n*	%
Getting circumcised makes me feel good because it means that I take care of my health.	10	1.4	178	25.4	400	57.1	107	15.3	5	0.7
Failing to get circumcised can increase my risk of getting HIV.	14	2.0	173	24.7	369	52.7	140	20.0	4	0.6
Failing to get circumcised can increase my risk of getting HPV.	18	2.6	172	24.6	417	59.6	87	12.4	6	0.9
Circumcision goes against my culture.	57	8.1	464	66.3	159	22.7	16	2.3	4	0.6
Getting circumcised will negatively affect my sexual performance.	101	14.6	447	64.4	115	16.6	31	4.5	6	0.9
Getting circumcised is painful.	18	2.6	175	25.2	409	58.9	91	13.1	7	1.0
Getting circumcised helps to protect me against disease even if I have unprotected sex.	23	3.3	182	26.2	384	54.9	106	15.3	5	0.7
Circumcision can prevent other serious health problems.	19	2.7	200	28.8	373	53.3	103	14.7	5	0.7
Getting circumcised is expensive.	64	9.2	434	62.4	158	22.7	39	5.6	5	0.7
Getting circumcised is embarrassing for me.	123	17.8	524	75.7	35	5.1	10	1.4	8	1.1
Getting circumcised might make potential sexual partners reluctant to have sex with me.	100	14.3	446	64.4	131	18.9	16	2.3	7	1.0
I do not have information about where to go to get a circumcision.	103	14.9	512	73.9	64	9.2	14	2.0	7	1.0
Getting circumcision is a good investment of my time for my health.	23	3.3	226	32.6	343	49.4	102	14.7	6	0.9
I do not know at what age it is necessary to have a circumcision.	75	10.8	521	75.2	88	12.7	9	1.3	7	1.0
I have not had a circumcision because when I go, I need to wait a long time to be seen.	136	19.6	521	75.1	31	4.5	6	0.9	6	0.9
My partner wants me to get a circumcision.	19	2.7	249	35.8	375	54.0	52	7.5	5	0.7
Circumcision can save my life.	11	1.6	195	28.1	362	52.1	127	18.3	5	0.7
I have not had a circumcision because I am afraid to attend a health centre that might advise me that I have another sexually transmitted disease.	38	6.3	444	74.0	102	17.0	16	2.7	100	14.3
I have not had circumcision because the health care centre is only open during hours when I cannot go.	66	9.5	597	86.1	27	3.9	3	0.4	7	1.0
I have not been circumcised because I am embarrassed to have a genital procedure.	60	8.7	520	75.3	104	15.1	7	1.0	9	1.3
Being circumcised does not protect me from getting HIV.	86	12.4	439	63.3	143	20.6	25	3.6	7	1.0
Circumcision exposes my foreskin to more infection.	97	14.0	532	76.8	57	8.2	7	1.0	7	1.0
I am not at risk for contracting HIV.	90	13.0	449	65.1	126	18.3	25	3.6	10	1.4
As I do not have a history of sexually transmitted diseases, it is very unlikely that I will get HIV.	90	13.0	479	69.2	93	13.4	29	4.2	9	1.3
If I am sterilised, I do not need a circumcision.	105	15.2	456	66.0	96	13.9	34	4.9	9	1.3
If I do not have symptoms of an STI, I do not need a circumcision.	86	12.4	474	68.4	98	14.1	35	5.1	7	1.0
If I do not have intercourse, I do not need a circumcision.	84	12.1	471	68.0	104	15.0	34	4.9	7	1.0

HIV, human immunodeficiency virus; HPV, human papilloma virus; STI, sexually transmitted infection.

Table 4 shows the association between different beliefs or attitudes and obtaining an MMC. People who had an MMC were more likely to believe in the positive health benefits and protection from HIV and HPV. They felt less at risk of contracting HIV, but they also believed that they were still protected during unprotected sex. People who had an MMC were more likely to believe partners might be reluctant to have sex with them. While seeing it as a good investment of their time, they were more likely to see it as costly, painful and time-consuming.

**TABLE 4:** Odds of medical male circumcision if you agree with the statement.

Variables	OR	95% CI	*p*
Getting circumcised makes me feel good because it means that I take care of my health.	62.9	27.3–144.8	< 0.001
Failing to get circumcised can increase my risk of getting HIV.	45.0	21.6–93.6	< 0.001
Failing to get circumcised can increase my risk of getting HPV.	22.5	12.9–39.4	< 0.001
Circumcision can save my life.	17.8	10.9–28.9	< 0.001
Getting circumcised might make potential sexual partners reluctant to have sex with me.	15.9	8.82–28.9	< 0.001
Getting circumcised helps to protect me against disease even when I have unprotected sex.	13.2	8.40–20.7	< 0.001
Circumcision can prevent other serious health problems.	10.5	6.99–15.9	< 0.001
Getting circumcision is a good investment of my time for my health.	8.35	5.78–12.1	< 0.001
Being circumcised does not protect me from getting HIV.	3.05	2.10–4.43	< 0.001
My partner wants me to get a circumcision.	2.94	2.14–4.04	< 0.001
Getting circumcised is painful.	2.89	2.04–4.11	< 0.001
I have not had a circumcision because when I go, I need to wait a long time to be seen.	1.49	0.76–2.92	0.247
I am not at risk of contracting HIV.	1.38	0.96–1.99	0.08
Getting circumcised is expensive.	1.27	0.91–1.77	< 0.001
Getting circumcised will negatively affect my sexual performance.	1.23	0.85–1.78	0.264
I do not know at what age it is necessary to have a circumcision.	0.69	0.45–1.06	0.093
Circumcision goes against my culture.	0.55	0.39–0.78	< 0.001
I do not have information about where to go to get a circumcision.	0.55	0.34–0.89	0.016
Getting circumcised is embarrassing for me.	0.52	0.28–0.99	0.046
I have not been circumcised because the health care centre is only open during hour when I cannot go.	0.48	0.22–1.05	0.066
Circumcision exposes my foreskin to more infection.	0.25	0.14–0.46	< 0.001
As I do not have a history of sexually transmitted diseases, it is very unlikely that I will get HIV.	0.15	0.09–0.24	< 0.001
I have not been circumcised because I am embarrassed to have a genital procedure.	0.10	0.05–0.18	< 0.001
If I am sterilised, I do not need a circumcision.	0.06	0.03–0.11	< 0.001
If I do not have intercourse, I do not need a circumcision.	0.06	0.03–0.12	< 0.001
If I do not have symptoms of an STI, I do not need a circumcision.	0.05	0.03–01.1	< 0.001

HIV, human immunodeficiency virus; OR, odds ratio, CI, confidence interval; HPV, human papilloma virus; STI, sexually transmitted infection.

People who had not had MMC were less informed and were more likely to see it as against their culture. They felt less at risk from Sexually transmitted infections (STIs) and thought that if they had no symptoms of STIs they did not need an MMC. They also felt less risk if they were sexually inactive and believed that if they were sterilised they did not need MMC. They were more likely to see it as embarrassing and to put them at risk of complications, such as infection of their foreskin.

Table 5 shows the results of the bivariate and multiple variable analyses for sociodemographic factors associated with MMC. In the bivariate analysis, all the variables were significantly associated with MMC, but in the multiple variable analysis only four factors remained. The analysis showed that being a student, attending a mainline Christian denomination, speaking an African language other than Zulu and being South African were associated with obtaining an MMC. Adjusted odds ratios are only presented for those variables that remained in the model.

**TABLE 5:** Bivariate and multivariable analysis of factors associated with medical male circumcision.

Variables	OR	95% CI	*p*	AOR	95% CI	*p*
**Age**	0.98	0.97–1.00	0.062	-	-	-
**Marital status**				-	-	-
Divorced	Reference			-	-	-
Single	3.04	1.18–7.85	0.022	-	-	-
Partner	2.20	0.83–5.82	0.114	-	-	-
Married or widowed	7.08	2.25–22.29	0.028	-	-	-
**Nationality**				-	-	-
Not South African	Reference			Reference		
South Africa	2.2	1.6–3.0	< 0.001	2.50	1.58–3.96	< 0.001
**Highest qualification**						
None or some primary	Reference			-	-	-
Primary	0.85	0.51–1.42	0.538	-	-	-
Some high school	1.77	1.07–2.92	0.026	-	-	-
High school	1.41	0.87–2.27	0.160	-	-	-
Higher education	1.17	0.62–2.22	0.633	-	-	-
**Religion**						
None	Reference			Reference		
Mainline Christian	4.67	2.49–8.77	< 0.001	2.83	1.39–5.78	0.004
Zionist	1.36	0.71–2.62	0.349	0.84	0.41–1.73	0.632
Not Christian	5.85	0.68–50.3	0.108	4.15	0.44–38.9	0.213
**Employment**						
Unemployed	Reference			Reference		
Employed	0.93	0.66–1.32	0.68	0.91	0.62–1.32	0.612
Self-employed	0.76	0.46–1.28	0.31	0.91	0.52–1.59	0.732
Informal	0.55	0.30–1.01	0.55	0.68	0.34–1.31	0.237
Student	10.1	3.93–26.2	< 0.001	6.29	2.29–17.26	< 0.001
**Home language**						
Zulu	Reference			Reference		
Xhosa	4.84	2.67–8.77	< 0.001	4.48	2.42–8.29	< 0.001
Ndebele	2.07	1.29–3.31	0.002	3.70	2.08–6.59	< 0.001
Siswati	2.68	1.33–5.42	0.06	3.47	1.65–7.28	< 0.001
Sepedi	7.88	3.83–16.2	< 0.001	6.82	3.14–14.8	< 0.001
Setswana	2.32	1.15–4.70	0.02	4.10	1.85–9.10	< 0.001
Sotho	1.75	0.86–3.46	0.108	3.14	1.39–7.09	0.006
Tonga	3.88	1.82–8.24	0.001	3.52	1.61–7.70	0.002
Venda	3.94	1.57–9.90	0.004	3.36	1.26–8.95	0.015
Other	1.05	0.60–1.85	0.866	2.50	1.23–5.09	0.011
**Address**						
Extension 13	Reference					
Extension 1	2.09	1.25–3.47	0.005	-	-	-
Extension 2	0.63	0.13–1.26	0.188	-	-	-
Extension 3	1.48	0.83–2.66	0.189	-	-	-
Extension 4	1.09	0.53–2.25	0.811	-	-	-
Extension 5	1.42	0.71–2.83	0.319	-	-	-
Extension 6	0.89	0.43–1.86	0.756	-	-	-
Extension 7	1.05	0.54–2.04	0.893	-	-	-
Extension 8	0.75	0.33–1.71	0.499	-	-	-
Extension 9	1.78	0.77–4.12	0.179	-	-	-
Extension 10	1.03	0.39–2.70	0.519	-	-	-
Extension 11	1.46	0.46–4.68	0.519	-	-	-
Extension 12	1.20	0.37–3.27	0.865	-	-	-

OR, odds ratio; CI, confidence interval; AOR, adjusted odds ratio.

## Discussion

**FIGURE 2:**
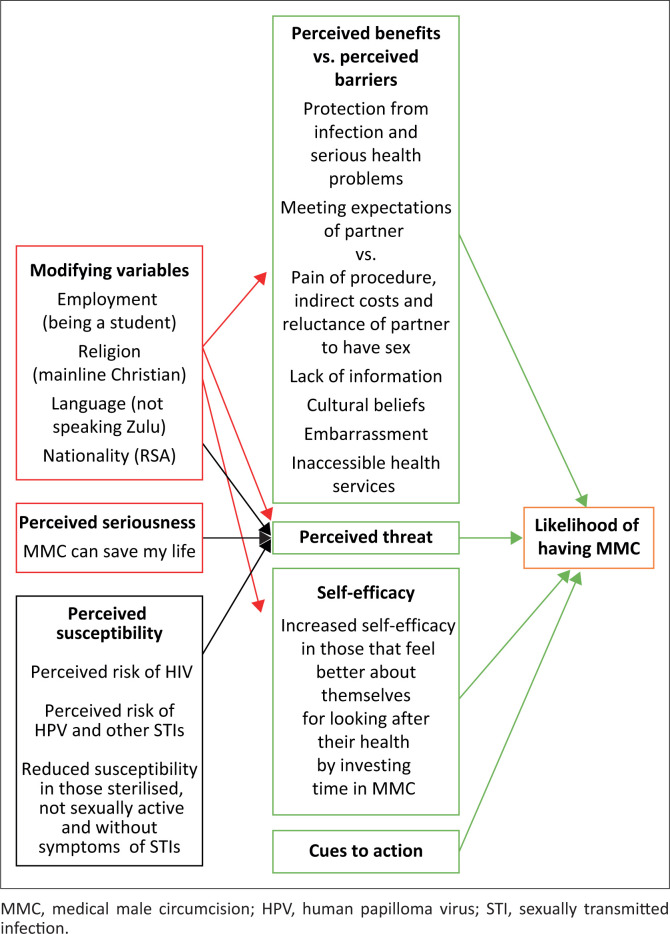
Summary of key findings in the health belief model.

The findings of the study are summarised using the health belief model in Figure 2. This model attempts to predict whether a person’s beliefs will translate into health-related behaviour or action. Motivation to undertake a health behaviour is seen as a balance between the perceived benefits, barriers and threats along with self-efficacy and cues to action.^20^ The perceived threat to the person is itself influenced by the perceived seriousness of the problem and the perceived susceptibility of the self to the problem. One’s perceptions and self-efficacy are also influenced by external modifying factors.

One key modifying factor was language, which is closely linked to culture. A survey, conducted in South Africa in 2008, also found that Zulus were the least likely to be circumcised.^21^ Traditional circumcision was practised among the Zulus up until the 19th century when the legendary King Shaka prohibited it, because it deprived him of young warriors for months at a time. However, King Zwelithini and political leadership in KwaZulu-Natal have recently endorsed medical circumcision,^22^ although it is likely that historical and cultural attitudes persist. Other ethnic groups, such as the AmaXhosa, continue to practise traditional circumcision and are likely to be more open towards medical circumcision.^21^

In this study, 41% of the men enrolled were foreign nationals, which illustrates the importance of this group in HIV prevention. The decreased chance of foreign nationals being circumcised may also be because of cultural issues. The largest group was from Zimbabwe, where it seems that attitudes are also against circumcision. A study from Mashonaland reported that circumcised men were perceived as ‘half a man’ and saw themselves as emasculated by the loss of their foreskins.^22^ In addition, the circumcision initiative is for South African citizens, and foreigners may not be eligible for a free service. The negative attitudes of some health care workers in government hospitals and clinics towards foreigners may also discourage access to circumcision.^21^

Other studies have also shown that members of mainline churches are more in support of circumcision (53%) than African traditional religions (27%) or Islam (0%).^23^ Mainline churches have been active in HIV education, have programmes that promote safe sex and members may be more exposed to information on circumcision.^23^ Catholic churches have not promoted condoms and are against religious circumcision, but are neutral when it comes to medical circumcision.^23^ With regard to religion, male circumcision tends to be far more prevalent among Muslims in all countries where Muslims are listed as a separate religious groups.^23^ However, in the community studied here, Christians were the majority.

Previous studies from KwaZulu-Natal reported that men presenting for MMC were young, living in urban townships, single, employed, more educated and sexually active.^24,25^ Our study was conducted in an urban area, but age and being single, employed or more educated were not associated with MMC. Being a student was a factor associated with MMC, and it is possible that students were more exposed to health promotion messages on HIV through higher education.^24^ A study from Lesotho, Mozambique, Swaziland and Zimbabwe showed that a greater proportion of men over 35 years were circumcised, compared to younger men. However, in our study, age was not a significant factor in the uptake of MMC.^26^

In a study from 2012, in four of nine provinces (Eastern Cape, Gauteng, KwaZulu-Natal and Mpumalanga) in South Africa, a high number of the participants (96%) had knowledge regarding MMC and found it acceptable (84%).^27^ Knowledge of the protective effect of male circumcision against HIV and reducing the risks for sexual partners was associated with the acceptability of male circumcision.^27^ In KwaZulu-Natal province, men also believed that MMC reduced the risks of herpes, chancroid, syphilis and penile cancer.^28^ In Lesotho, MMC was also associated with the belief that it improved health, reduced the risk of HIV and STIs, improved penile hygiene and sexual pleasure and enhanced social prestige. Other studies have also found an association with women encouraging their partners to have MMC.^29^

In our study, those who had MMC also held strong beliefs about the health benefits and prevention of infectious diseases such as HIV. On the other hand, those who had MMC were also more likely to believe that they were safe during unprotected sex. While MMC reduces transmission, it does not fully protect from HIV, HPV and other infections, and barrier methods such as condoms are still needed. This false sense of security among those with MMC also needs to be addressed in health education.

As this was a case-control study, factors are associated with the outcome, but a cause and effect relationship cannot be proven. Nevertheless, the relationship between factors can be logically attributed, even though not proven; for example, in those with MMC it is likely that the belief in the protective effect of MMC was instrumental in the decision to have the procedure.

The control group was selected from the shopping mall, and during the coronavirus disease 2019 (COVID-19) pandemic and lockdown, it is likely that unemployment led many people to shop locally and not travel to the mall. Two other malls were also available in the area. This could have introduced a selection bias, although employment versus unemployment was not found to be a significant factor. The case group was selected from a private practice offering free MMC sponsored by the government. Nevertheless, it is possible that those choosing to have the MMC at the private practice were different from those choosing to have it done at a public sector facility.

A few of the statements that participants responded to were worded in a way that implied which group the person should belong to. It would have been better to phrase all the statements more neutrally for both groups. For example, the statement ‘I have not been circumcised because I am embarrassed to have a genital procedure’ speaks more directly about the control group.

Diepsloot is a metropolitan township in Johannesburg, with a mix of different people typical of Gauteng. The mix of cultures, languages and population groups would however be very different from other provinces such as the Western Cape or KwaZulu-Natal. Likewise, the urban setting cannot be generalised to more rural areas. Hence, the results may not be generalised to other districts in South Africa, although it is likely that many of these factors would also be identified elsewhere.

The health belief model helps to focus attention on key issues that health promotion should focus on in this community. The perceived susceptibility to and seriousness of HIV infection should be addressed and, in particular, misinformation regarding the protection offered by sterilisation as well as the absence of risk if there are no symptoms of an STI. The value of MMC to reduce future risk, even if one is not currently sexually active, should also be addressed.

Culture and traditions can act as barriers to the acceptability of MMC.^28^ It is important that health programmes such as MMC engage with indigenous practices and beliefs to reap the maximum benefits from such programmes. This should also be extended to healthcare providers, as some beliefs are held by healthcare workers and may influence how they advocate for such health programmes.^28^

Further attention should be given to understanding the perceptions of Zulu-speaking community members and what issues need to be addressed to overcome resistance to MMC. Attention may also need to be given to migrants in the community and barriers to MMC, such as the cost of the procedure and making health services more migrant-friendly.

Health promotion messages should be tailored to reach groups less likely to accept MMC, such as those attending Zionist and other Christian denominations. Engaging religious and faith-based leaders and understanding the theological issues involved may assist with this.^29^ Similarly, the workplace can be an effective place for health promotion, and MMC could be one of the issues addressed.^30^ Local media that reaches the whole community, such as radio and newspapers, may also be helpful in reaching the community.

Therefore, qualitative studies could explore issues behind some of the factors identified and provide insights useful to health promotion. Factors such as being a student, attending a mainline Christian denomination, speaking Zulu and being a foreigner could be further explored.

## Conclusions

People who had MMC were more likely to believe in the positive health benefits and protection from HIV and HPV. Culture and traditions were the factors against MMC. Being a student, attending a mainline Christian domination, speaking an African language other than Zulu and being a South African were associated with obtaining MMC.

The health belief model helps to focus attention on the key issues that health promotion and disease prevention should address in this community. The perceived susceptibility to and seriousness of HIV infection should be addressed and, in particular, misinformation regarding the protection offered by sterilisation as well as the absence of risk if there are no symptoms of an STI. Men with MMC believed that they were fully protected during unprotected sex, and this also needs to be addressed.
